# Mutation and Chaos in Nonlinear Models of Heredity

**DOI:** 10.1155/2014/835069

**Published:** 2014-07-21

**Authors:** Nasir Ganikhodjaev, Mansoor Saburov, Ashraf Mohamed Nawi

**Affiliations:** Department of Computational & Theoretical Sciences, Faculty of Sciences, International Islamic University Malaysia, P.O. Box 141, 25710 Kuantan, Pahang, Malaysia

## Abstract

We shall explore a nonlinear discrete dynamical system that naturally occurs in population systems to describe a transmission of a trait from parents to their offspring. We consider a Mendelian inheritance for a single gene with three alleles and assume that to form a new generation, each gene has a possibility to mutate, that is, to change into a gene of the other kind. We investigate the derived models and observe chaotic behaviors of such models.

## 1. Introduction

Recently, chaotic dynamical systems become very popular in science and engineering. Besides the original definition of the Li-Yorke chaos [1], there have been various definitions for “chaos” in the literature, and the most often used one is given by Devaney [2]. Altshough there is no universal definition for chaos, the essential feature of chaos is sensitive dependence on initial conditions so that the eventual behavior of the dynamics is unpredictable. The theory and methods of chaotic dynamical systems have been of fundamental importance not only in mathematical sciences, but also in physical, engineering, biological, and even economic sciences. In this paper, a chaos would be understood in the sense of Li-Yorke [3, 4] (the precise definition will be given in the next section).

In this paper, we introduce and examine a family of nonlinear discrete dynamical systems that naturally occurs to describe a transmission of a trait from parents to their offspring. Here, we shall present some essential analytic and numerical results on dynamics of such models.

In [5], it was presented an approach to the dynamics at the cellular scale in which cells can progress, namely, modify their biological expression and mutate within Darwinian-type selective processes, out of the interaction with other cells. A heterogeneous distribution among cells produces mutations and selections generated by net destructive and/or proliferative events [5]. In this event, all living systems are evolutionary: birth processes can generate individuals that fit better the outer environment, which in turn generates new ones better and better fitted [5]. One can refer to [5–8] for the general information about mathematical models of complex systems (including mutations and selections). In this paper, we are presenting a mathematical model of the evolution of the percentage of different alleles of a given trait after the mutation process.

As the first example, we consider a Mendelian inheritance of a single gene with two alleles **A** and **a** (see [9]). Let an element **x** = (*x*_1_, *x*_2_) represent a gene pool for a population; that is, *x*_1_, *x*_2_ are the percentage of the population which carries the alleles **A** and **a**, respectively. For the convenience, we express it as a linear combination of the alleles **A** and **a**

(1) $$\begin{array}{c} x=x_{1}A+x_{2}a, \end{array}$$

where, 0 ≤ *x*_1_,  *x*_2_ ≤ 1 and *x*_1_ + *x*_2_ = 1. The rules of the Mendelian inheritance indicate that the next generation has the following form:

(2) $$\begin{array}{c} x^{\prime }=x_{1}^{\prime }A+x_{2}^{\prime }a, \end{array}$$

where

(3) $$\begin{array}{c} x_{1}^{\prime }=P_{AA,A}x_{1}^{2}+2P_{Aa,A}x_{1}x_{2}+P_{aa,A}x_{2}^{2},\\ x_{2}^{\prime }=P_{AA,a}x_{1}^{2}+2P_{Aa,a}x_{1}x_{2}+P_{aa,a}x_{2}^{2}. \end{array}$$

Here, *P*_*AA*,*A*_ (resp., *P*_*AA*,*a*_) is the probability that the child receives the allele **A** (resp., **a**) from parents with the allele **A**; *P*_*Aa*,*A*_ (resp., *P*_*Aa*,*a*_) is the probability that the child receives the allele **A** (resp., **a**) from parents with the alleles **A** and **a**, respectively; and *P*_*aa*,*A*_ (resp., *P*_*aa*,*a*_) is the probability that the child receives the allele **A** (resp., **a**) from parents with allele **a**. It is evident that

(4) $$\begin{array}{c} P_{\ldots ,A}+P_{\ldots ,a}=1,\;\;P_{Aa,A}=P_{aA,A},\\ P_{Aa,a}=P_{aA,a},\;\;x_{1}^{\prime }+x_{2}^{\prime }=1. \end{array}$$

Thus, the evolution (3) is a nonlinear dynamical system acting on the one dimensional symplex

(5) $$\begin{array}{c} S^{1}=\left\{\left(x_{1},x_{2}\right)\in R^{2}:x_{1},x_{2}\geq 0,x_{1}+x_{2}=1\right\} \end{array}$$

which describes the distribution of the next generation which carries the alleles **A** and **a**, respectively, if the distribution of the current generation is known.

Recall that in the simple Mendelian inheritance case, that is, *P*_*AA*,*A*_ = *P*_*aa*,*a*_ = 1 and *P*_*AA*,*a*_ = *P*_*aa*,*A*_ = 0, the dynamical system (3) has the following form:

(6) $$\begin{array}{c} x_{1}^{\prime }=x_{1}^{2}+2P_{Aa,A}x_{1}x_{2},\\ x_{2}^{\prime }=2P_{Aa,a}x_{1}x_{2}+x_{2}^{2}. \end{array}$$

We assume that prior to a formation of a new generation each gene has a possibility to mutate, that is, to change into a gene of the other kind. Specifically, we suppose that for each gene the mutation **A** → **a** occurs, with probability *α*, and **a** → **A** occurs with probability *β*. Moreover, we assume that “*the mutation occurs if and only if both parents have the same allele*.” Then, we have that *P*_*AA*,*a*_ = *α*,  *P*_*aa*,*A*_ = *β*, *P*_*AA*,*A*_ = 1 − *α*,  *P*_*aa*,*a*_ = 1 − *β* and the dynamical system (3) has the following form:

(7) $$\begin{array}{c} V:\{\begin{array}{cc} x_{1}^{\prime }=\left(1-\alpha \right)x_{1}^{2}+2P_{Aa,A}x_{1}x_{2}+\beta x_{2}^{2}, & \\ x_{2}^{\prime }=\alpha x_{1}^{2}+2P_{Aa,a}x_{1}x_{2}+\left(1-\beta \right)x_{2}^{2}. & \end{array} \end{array}$$

An operator *V* : *S* ^1^ → *S* ^1^ given by (7) is called* a quadratic stochastic operator* [10]. The name “*stochastic*” can be justified if we consider the simplex as a set of all probability distributions of the finite set, so that, the operator (7) maps a probability distribution to a probability distribution.

We introduce some standard terms in the theory of a discrete dynamical system *V* : *X* → *X*. A sequence {**x** ^(*n*)^}_*n*=0_^*∞*^, where **x** ^(*n*)^ = *V*(**x** ^(*n*−1)^), is called a trajectory of *V* starting from an initial point **x** ^0^. Recall that a point **x** is called a fixed point of *V* if *V*(**x**) = **x**. We denote a set of all fixed points by Fix⁡(*V*). A dynamical system *V* is called regular if a trajectory {**x** ^(*n*)^}_*n*=0_^*∞*^ converges for any initial point **x**. Note that if  *V* is regular, then limiting points of *V* are fixed points of *V*. Thus, in a regular system, the fixed point of dynamical system describes a long run behavior of the trajectory of *V* starting from any initial point. The biological treatment of the regularity of dynamical system *V* is rather clear: in a long run time, the distribution of species in the next generation coincide with distribution of species in the current generation, that is, stable.

A fixed point set and an omega limiting set of quadratic stochastic operators (QSO) were deeply studied in [11–16] and quadratic stochastic operators (QSO) play an important role in many applied problems [17, 18]. In [10], it was given a long self-contained exposition of recent achievements and open problems in the theory of quadratic stochastic operators.

**Definition 1 .**

A dynamical system *V* : *X* → *X* is said to be ergodic if the limit

(8) $$\begin{array}{c} \underset{n\to \infty }{\lim}\frac{1}{n}\overset{n-1}{\underset{k=0}{\sum }}V^{k}\left(x\right) \end{array}$$

exists for any **x** ∈ *X*.

Based on some numerical calculations, Ulam has conjectured [19] that any QSO acting on the finite dimensional simplex is ergodic. However, Zakharevich showed [20] that, in general, Ulam's conjecture is false. Namely, Zakharevich showed that the following QSO *V*_0_ : *S* ^2^ → *S* ^2^ is not ergodic:

(9) $$\begin{array}{c} V_{0}:\{\begin{array}{cc} x_{1}^{\prime }=x_{1}^{2}+2x_{1}x_{2}, & \\ x_{2}^{\prime }=x_{2}^{2}+2x_{2}x_{3}, & \\ x_{3}^{\prime }=x_{3}^{2}+2x_{3}x_{1}. & \end{array} \end{array}$$

In [21], Zakharevich's result was generalized in the class of Volterra QSO.

We define the *k*th order Cesaro mean by the following formula:

(10) $$\begin{array}{c} \text{Ce}{\text{s}}_{k}^{\left(n\right)}\left(x,V\right)=\frac{1}{n}\overset{n-1}{\underset{i=0}{\sum }}\text{Ce}{\text{s}}_{k-1}^{\left(i\right)}\left(x,V\right), \end{array}$$

where *k* ≥ 1 and Ces_0_^(*n*)^(**x**, *V*) = *V* ^*n*^(**x**). It is clear that the first order Cesaro mean Ces_1_^(*n*)^(**x**, *V*) is nothing but (1/*n*)∑_*i*=0_^*n*−1^ *V* ^*i*^(**x**). Based on these notations, Zakharevich's result says that the first order Cesaro mean {Ces_1_^(*n*)^(**x**,*V*_0_)}_*n*=0_^*∞*^ of the trajectory of the operator *V*_0_ given by (9) diverges for any initial point except fixed points. Surprisingly, in [22], it was proven that any order Cesaro mean {Ces_*k*_^(*n*)^(**x**,*V*_0_)}_*n*=0_^*∞*^, for any *k* ∈ *N*, of the trajectory of the operator *V*_0_ diverges for any initial point except fixed points. This leads to a conclusion that the operator *V*_0_ might have unpredictable behavior. In fact, in [23], it was proven that the operator *V*_0_ exhibits the Li-Yorke chaos. It is worth pointing out that some strange properties of Volterra QSO were studied in [24, 25].

In the literature, all examples of nonergodic QSO have been found in the class of Volterra QSO (see [10, 20, 21]). Based on these examples, the Ulam conjecture was modified as follows:* any non Volterra QSO acting on the finite dimensional simplex is ergodic, that is, operators having chaotic behavior can be only found among Volterra QSO.* However, in this paper, we are aiming to present the continual family of nonergodic and chaotic QSO which are non Volterra QSO.

Note that if QSO is regular, then it is ergodic. However, the reverse implication is not always true. It is known that the dynamical system (7) is either regular or converges to a periodic-2 point [26]. Therefore, in 1D simplex, any QSO is ergodic. In other words, the evolution of a mutation in population system having* a single gene with two alleles* always exhibits an ergodic behavior (or almost regular or almost stable). It is of independent interest to study the evolution of a mutation in population system having* a single gene with three alleles*. In the next section, we consider an inheritance of a single gene with three alleles **a**_1_, **a**_2_, and **a**_3_ and show that a nonlinear dynamical system corresponding to the mutation exhibits a nonergodic and Li-Yorke chaotic behavior.

## 2. Inheritance for a Single Gene with Three Alleles

In this section, we shall derive a mathematical model of an inheritance of a single gene with three alleles.

As it was in the previous section, an element **x** represents a linear combination **x** = *x*_1_ **a**_1_ + *x*_2_ **a**_2_ + *x*_3_ **a**_3_ of the alleles **a**_1_, **a**_2_, and **a**_3_ in which the following conditions are satisfied 0 ≤ *x*_1_, *x*_2_, *x*_3_ ≤ 1 and *x*_1_ + *x*_2_ + *x*_3_ = 1, that is, *x*_1_, *x*_2_, *x*_3_ are the percentages of the population which carry the alleles **a**_1_, **a**_2_, and **a**_3_ respectively.

We assume that prior to a formation of a new generation each gene has a possibility to mutate, that is, to change into a gene of the other kind. We assume that the mutation occurs if* both parents have the same alleles*. Specifically, we will consider two types of the simplest mutations; assume that

1. mutations **a**_1_ → **a**_2_, **a**_2_ → **a**_3_, and **a**_3_ → **a**_1_ occur with probability *α*;
2. mutations **a**_1_ → **a**_3_, **a**_3_ → **a**_2_, and **a**_2_ → **a**_1_ occur with probability *α*.

In this case, the corresponding dynamical systems are defined on the two-dimensional simplex

(11) S2={x=(x1,x2,x3)∈R3: x1≥0,x2≥0,x3≥0,x1+x2+x3=1}.

In the first mutation, we have

(12) $$\begin{array}{c} V_{\alpha }:\{\begin{array}{cc} x_{1}^{\prime }=\left(1-\alpha \right)x_{1}^{2}+2x_{1}x_{2}+\alpha x_{2}^{2}, & \\ x_{2}^{\prime }=\left(1-\alpha \right)x_{2}^{2}+2x_{2}x_{3}+\alpha x_{3}^{2}, & \\ x_{3}^{\prime }=\left(1-\alpha \right)x_{3}^{2}+2x_{3}x_{1}+\alpha x_{1}^{2}. & \end{array} \end{array}$$

In the second mutation, we have

(13) $$\begin{array}{c} W_{\alpha }:\{\begin{array}{cc} x_{1}^{\prime }=\left(1-\alpha \right)x_{1}^{2}+2x_{1}x_{2}+\alpha x_{3}^{2}, & \\ x_{2}^{\prime }=\left(1-\alpha \right)x_{2}^{2}+2x_{2}x_{3}+\alpha x_{1}^{2}, & \\ x_{3}^{\prime }=\left(1-\alpha \right)x_{3}^{2}+2x_{3}x_{1}+\alpha x_{2}^{2}. & \end{array} \end{array}$$

Let us first recall the definition of the Li-Yorke chaos [1, 3, 4].

**Definition 2 .**

Let (*X*, *d*) be a metric space. A continuous map *V* : *X* → *X* is called* Li-Yorke chaotic* if there exists an uncountable subset *S* ⊂ *X* such that for every pair (*x*, *y*) ∈ *S* × *S* of distinct points, we have that

(14) $$\begin{array}{c} \underset{n\to \infty }{\mathrm{liminf}}\;d\left(V^{\left(n\right)}\left(x\right),V^{\left(n\right)}\left(y\right)\right)=0,\\ \underset{n\to \infty }{\mathrm{limsup}}\;d\left(V^{\left(n\right)}\left(x\right),V^{\left(n\right)}\left(y\right)\right)>0. \end{array}$$

In this case, *S* is* a scrambled set* and (*x*, *y*) ∈ *S* × *S* is* a Li-Yorke pair*.

Let us turn to the discussion of operators *V*_*α*_, *W*_*α*_ given by (12) and (13), respectively. In both cases, if *α* = 0, that is, if a mutation does not occur, then dynamical systems (12) and (13) coincide with Zakharevich's operator (9). As we already mentioned that Zakharevich's operator exhibits the Li-Yorke chaos [23].

Let *α* = 1. In the first case, the operator *V*_1_ is a permutation of Zakharevich's operator (9). Therefore, the operator *V*_1_ is nonergodic and does exhibit the Li-Yorke chaotic behavior [22, 23, 27]. In the second case, the operator *W*_1_ is a permutation of the regular operator which was studied in [11]. By applying the same method which was used in [11], we may easily show that the operator *W*_1_ is also regular.

It is easy to check that *V*_*α*_ = (1 − *α*)*V*_0_ + *αV*_1_ and *W*_*α*_ = (1 − *α*)*W*_0_ + *αW*_1_.

This means that, in the first case, the evolution operator *V*_*α*_ is a convex combination of two Li-Yorke chaotic operators *V*_0_, *V*_1_, meanwhile, in the second case, the evolution operator *W*_*α*_ is a convex combination of the Li-Yorke chaotic and regular operators *W*_0_, *W*_1_. These operators *V*_*α*_, *W*_*α*_ were not studied in [11, 27]. It is of independent interest to study the dynamics of operators *V*_*α*_ and *W*_*α*_. The reason is that, in the first case, the convex combination presents a transition from one chaotic biological system to another chaotic biological system (we shall see in the next section that, in some sense, their dynamics are opposite each other); meanwhile, in the second case, the convex combination presents a transition from the ordered biological system to the chaotic biological system. In the next section, we are going to present some essential analytic and numerical results on dynamics of the operators *V*_*α*_ and *W*_*α*_ given by (12) and (13), respectively.

## 3. Attractors: Analytic and Numerical Results

### 3.1. Analytic Results on Dynamics of *V*_*α*_

We are aiming to present some analytic results on dynamics of *V*_*α*_ : *S* ^2^ → *S* ^2^:

(15) $$\begin{array}{c} V_{\alpha }:\{\begin{array}{cc} x_{1}^{\prime }=\left(1-\alpha \right)x_{1}^{2}+2x_{1}x_{2}+\alpha x_{2}^{2}, & \\ x_{2}^{\prime }=\left(1-\alpha \right)x_{2}^{2}+2x_{2}x_{3}+\alpha x_{3}^{2}, & \\ x_{3}^{\prime }=\left(1-\alpha \right)x_{3}^{2}+2x_{3}x_{1}+\alpha x_{1}^{2}, & \end{array} \end{array}$$

where *V*_*α*_(*x*) = *x*′ = (*x*_1_′, *x*_2_′, *x*_3_′) and 0 < *α* < 1. As we already mentioned, this operator can be written in the following form: *V*_*α*_ = (1 − *α*)*V*_0_ + *αV*_1_ for any 0 < *α* < 1, where

(16) $$\begin{array}{c} V_{0}:\{\begin{array}{cc} x_{1}^{\prime }=x_{1}^{2}+2x_{1}x_{2}, & \\ x_{2}^{\prime }=x_{2}^{2}+2x_{2}x_{3}, & \\ x_{3}^{\prime }=x_{3}^{2}+2x_{3}x_{1}, & \end{array}\\ V_{1}:\{\begin{array}{cc} x_{1}^{\prime }=x_{2}^{2}+2x_{1}x_{2}, & \\ x_{2}^{\prime }=x_{3}^{2}+2x_{2}x_{3}, & \\ x_{3}^{\prime }=x_{1}^{2}+2x_{3}x_{1}. & \end{array} \end{array}$$

Let

(17) $$\begin{array}{c} P=\left(\begin{array}{ccc} 0 & 1 & 0\\ 0 & 0 & 1\\ 1 & 0 & 0 \end{array}\right) \end{array}$$

be a permutation matrix. The proofs of the following results are straightforward.

**Proposition 3 .**

Let *V*_*α*_ : *S* ^2^ → *S* ^2^ be the evolution operator given by (15), where *α* ∈ (0,1). Let Fix(*V*_*α*_) and *ω*(*x* ^0^) be sets of fixed points and omega limiting points of  *V*_*α*_, respectively. Then the following statements hold true.

- Operators *P* and *V*_*α*_ are commutative, that is, *P*∘*V*_*α*_ = *V*_*α*_∘*P*.
- If *x* ∈ Fix⁡(*V*_*α*_) then *Px* ∈ Fix⁡(*V*_*α*_).
- If Fix⁡(*V*_*α*_) is a finite set then |Fix⁡(*V*_*α*_)| ≡ 1(mod⁡3).
- One has that *P*(*ω*(*x* ^0^)) = *ω*(*Px* ^0^), for any *x* ^0^ ∈ *S* ^2^.

We are aiming to study the fixed point set Fix⁡(*V*_*α*_), where *α* ∈ (0,1). It is worth mentioning that Fix⁡(*V*_0_) = {*e*_1_, *e*_2_, *e*_3_, *C*} and Fix⁡(*V*_1_) = {*C*}, where *e*_1_ = (1,0, 0), *e*_2_ = (0,1, 0), and *e*_3_ = (0,0, 1) are vertices of the simplex *S* ^2^ and *C* = (1/3, 1/3, 1/3) is a center of the simplex *S* ^2^.

Recall that a fixed point *x* ^0^ ∈ Fix⁡(*V*_*α*_) is nondegenerate [18] if and only if the following determinant is nonzero at the fixed point *x* ^0^:

(18) $$\begin{array}{c} \left|\begin{array}{ccc} \frac{\partial x_{1}^{\prime }}{\partial x_{1}}-1 & \frac{\partial x_{1}^{\prime }}{\partial x_{2}} & \frac{\partial x_{1}^{\prime }}{\partial x_{3}}\\ \frac{\partial x_{2}^{\prime }}{\partial x_{1}} & \frac{\partial x_{2}^{\prime }}{\partial x_{2}}-1 & \frac{\partial x_{2}^{\prime }}{\partial x_{3}}\\ 1 & 1 & 1 \end{array}\right|\neq 0 \end{array}$$

**Proposition 4 .**

Let *V*_*α*_ : *S* ^2^ → *S* ^2^ be the evolution operator given by (15), where *α* ∈ (0,1). Let *C* = (1/3, 1/3, 1/3) be a center of the simplex *S* ^2^. Then the following statements hold true.

- All fixed points are nondegenerate.
- One has that *Fix*(*V*_*α*_) = {*C*} for any *α* ∈ (0,1).

**Proof**

(i) Let *x* ∈ Fix⁡(*V*_*α*_) be a fixed point. One can easily check that

(19) |∂x1′∂x1−1∂x1′∂x2∂x1′∂x3∂x2′∂x1∂x2′∂x2−1∂x2′∂x3111| =4(1−α+α2)(x1x2+x1x3+x2x3)+2α−1.

If 1/2 ≤ *α* < 1, then the above expression is positive. Therefore, all fixed points are nondegenerate.

Let 0 < *α* < 1/2. In this case, the above expression is equal to zero if and only if *x*_1_ *x*_2_ + *x*_1_ *x*_3_ + *x*_2_ *x*_3_ = (1 − 2*α*)/(4(1 − *α* + *α* ^2^)). Since *x*_1_ + *x*_2_ + *x*_3_ = 1, we have that *x*_1_^2^ + *x*_2_^2^ + *x*_3_^2^ = (1 + 2*α* ^2^)/(2(1 − *α* + *α* ^2^)).

Without loss of generality, we may assume that *x*_1_ ≥ max⁡{*x*_2_, *x*_3_} (See Proposition 3(i)). Let *x*_2_ ≥ *x*_3_. Since *x* ∈ Fix⁡(*V*_*α*_), we have that *x*_2_ = (1 − *α*)*x*_2_^2^ + 2*x*_2_ *x*_3_ + *αx*_3_^2^. We then obtain that

(20) 1+2α22(1−α+α2) =x12+x22+x32 =x12+[(1−α)(x22+2x2x3)+α(x32+2x2x3)]2+x32 ≤x12+[(1−α)x2+αx3]2+x32 <x12+x22+x32=1+2α22(1−α+α2).

This is a contradiction. In a similar way, one can have a contradiction whenever *x*_3_ ≥ *x*_2_. This shows that, in the case 0 < *α* < 1/2, all fixed points are nondegenerate.

(ii) We shall show that Fix⁡(*V*_*α*_) = {*C*}. The simple calculation shows that *C* ∈ Fix⁡(*V*_*α*_). It is clear that *V*_*α*_(∂*S* ^2^) ⊂ int⁡*S* ^2^. This means that the operator *V*_*α*_ does not have any fixed point on the boundary ∂*S* ^2^ of the simplex *S* ^2^, that is, Fix⁡(*V*_*α*_)∩∂*S* ^2^ = *∅*. Moreover, all fixed points are nondegenerate. Due to Theorem 8.1.4 in [18], |Fix⁡(*V*_*α*_)| should be odd. On the other hand, due to Corollary 8.1.7 in [18], one has that |Fix⁡(*V*_*α*_)| ≤ 4. In Proposition 3, (iii) yields that |Fix⁡(*V*_*α*_)| = 1. Therefore, we get that Fix⁡(*V*_*α*_) = {*C*}.

A local behavior of the fixed point *C* = (1/3, 1/3, 1/3) is as follows.

**Proposition 5 .**

Let *V*_*α*_ : *S* ^2^ → *S* ^2^ be the evolution operator given by (15), where *α* ∈ (0,1). Then the following statements hold true.

- If *α* ≠ 1/2, then the fixed point *C* = (1/3, 1/3, 1/3) is repelling.
- If *α* = 1/2, then the fixed point *C* = (1/3, 1/3, 1/3) is nonhyperbolic.

**Proof**

It is worth mentioning that, since *x*_1_ + *x*_2_ + *x*_3_ = 1, the spectrum of the Jacobian matrix of the operator *V*_*α*_ : *S* ^2^ → *S* ^2^ at the fixed point *C* = (1/3, 1/3, 1/3) must be calculated as follows:

(21) $$\begin{array}{c} \left|\begin{array}{ccc} \frac{\partial x_{1}^{\prime }}{\partial x_{1}}-\lambda & \frac{\partial x_{1}^{\prime }}{\partial x_{2}} & \frac{\partial x_{1}^{\prime }}{\partial x_{3}}\\ \frac{\partial x_{2}^{\prime }}{\partial x_{1}} & \frac{\partial x_{2}^{\prime }}{\partial x_{2}}-\lambda & \frac{\partial x_{2}^{\prime }}{\partial x_{3}}\\ 1 & 1 & 1 \end{array}\right|=0. \end{array}$$

After simple algebra, we have that Spec$(V_{\alpha })=\left\{\lambda_{\pm }=1-\alpha \pm i(\sqrt{3}/3)(1+\alpha )\right\}$. It is clear that $|\lambda_{\pm }|=\sqrt{1+{\left(2\alpha -1\right)}^{2}/3}$. Consequently, if *α* ≠ 1/2, then the fixed point *C* = (1/3, 1/3, 1/3) is repelling and if *α* = 1/2, then the fixed point *C* = (1/3, 1/3, 1/3) is nonhyperbolic. This completes the proof.

We shall separately study two cases *α* ≠ 1/2 and *α* = 1/2.

**Theorem 6 .**

Let *V*_*α*_ : *S* ^2^ → *S* ^2^ be the evolution operator given by (15), where *α* ≠ 1/2. Then *ω*_*V*_*α*__(*x* ^0^) ⊂ int*S* ^2^ is an infinite compact subset, for any *x* ^0^ ≠ *C*.

**Proof**

Let *α* ≠ 1/2. Since *V*_*α*_ is continuous and *V*_*α*_(*S* ^2^) ⊂ int⁡*S* ^2^, an omega limiting set *ω*(*x* ^0^) is a nonempty compact set and *ω*(*x* ^0^) ⊂ int⁡*S* ^2^, for any *x* ^0^ ≠ *C*. We want to show that *ω*(*x* ^0^) is infinite, for any *x* ^0^ ≠ *C*. Since *C* is repelling, we have that *C* ∉ *ω*(*x* ^0^). Let us pick up any point x* ∈ *ω*(*x* ^0^) from the set *ω*(*x* ^0^). Since the operator *V*_*α*_ does not have any periodic point, the trajectory {*V*_*α*_^(*n*)^(x*)}_*n*=1_^*∞*^ of the point x* is infinite. Since *V*_*α*_ is continuous, we have that {*V*_*α*_^(*n*)^(x*)}_*n*=1_^*∞*^ ⊂ *ω*(*x* ^0^). This shows that *ω*(*x* ^0^) is infinite for any *x* ^0^ ≠ *C*.

**Remark 7 .**

It is worth mentioning that the sets of omega limiting points *ω*_*V*_0__(*x* ^0^) and *ω*_*V*_1__(*x* ^0^) of the operators *V*_0_ and *V*_1_ are infinite. However, unlike the operator *V*_*α*_, we have inclusions *ω*_*V*_0__(*x* ^0^)⊂∂*S* ^2^ and *ω*_*V*_1__(*x* ^0^)⊂∂*S* ^2^. Moreover, both operators *V*_0_ and *V*_1_ are nonergodic [20, 27].

Numerically, we shall see in the next section that the evolution operator *V*_*α*_ : *S* ^2^ → *S* ^2^ given by (15), where *α* ≠ 1/2, has the following properties.

- The operator *V*_*α*_ is nonergodic.
- The operator *V*_*α*_ exhibits the Li-Yorke chaos.

Now, we shall study the case *α* = 1/2. The operator *V*_1/2_ : *S* ^2^ → *S* ^2^ takes the following form

(22) $$\begin{array}{c} V_{1/2}:\{\begin{array}{cc} x_{1}^{\prime }=\frac{1}{2}x_{1}^{2}+2x_{1}x_{2}+\frac{1}{2}x_{2}^{2}\text{,} & \\ x_{2}^{\prime }=\frac{1}{2}x_{2}^{2}+2x_{2}x_{3}+\frac{1}{2}x_{3}^{2}, & \\ x_{3}^{\prime }=\frac{1}{2}x_{3}^{2}+2x_{3}x_{1}+\frac{1}{2}x_{1}^{2}. & \end{array} \end{array}$$

In this case, the fixed point *C* = (1/3, 1/3, 1/3) is nonhyperbolic and the spectrum of the Jacobian matrix of the operator *V*_1/2_ at the fixed point *C*, calculated by (18), is $Sp(J(C))=\left\{\left(1\pm \sqrt{3}i\right)/2\right\}$.

Let us define the following sets:

(23) $$\begin{array}{c} l_{1}=\left\{x\in S^{2}:x_{2}=x_{3}\right\},\;\;l_{2}=\left\{x\in S^{2}:x_{1}=x_{3}\right\},\\ l_{3}=\left\{x\in S^{2}:x_{1}=x_{2}\right\},\\ S_{1}=\left\{x\in S^{2}:x_{1}\geq x_{2}\geq x_{3}\right\},\\ S_{2}=\left\{x\in S^{2}:x_{1}\geq x_{3}\geq x_{2}\right\},\\ S_{3}=\left\{x\in S^{2}:x_{3}\geq x_{1}\geq x_{2}\right\},\\ S_{4}=\left\{x\in S^{2}:x_{3}\geq x_{2}\geq x_{1}\right\},\\ S_{5}=\left\{x\in S^{2}:x_{2}\geq x_{3}\geq x_{1}\right\},\\ S_{6}=\left\{x\in S^{2}:x_{2}\geq x_{1}\geq x_{3}\right\}. \end{array}$$

**Proposition 8 .**

We have the following cycles:

- $l_{1}\overset{V_{1/2}}{\to }l_{2}\overset{V_{1/2}}{\to }l_{3}\overset{V_{1/2}}{\to }l_{1}$;
- $S_{1}\overset{V_{1/2}}{\to }S_{2}\overset{V_{1/2}}{\to }S_{3}\overset{V_{1/2}}{\to }S_{4}\overset{V_{1/2}}{\to }S_{5}\overset{V_{1/2}}{\to }S_{6}\overset{V_{1/2}}{\to }S_{1}$;

**Proof**

Let *V*_1/2_ be an operator given by (22). One can easily check that

(24) $$\begin{array}{c} x_{1}^{\prime }-x_{2}^{\prime }=\left(x_{1}-x_{3}\right)\frac{1+3x_{2}}{2},\\ x_{1}^{\prime }-x_{3}^{\prime }=\left(x_{2}-x_{3}\right)\frac{1+3x_{1}}{2},\\ x_{2}^{\prime }-x_{3}^{\prime }=\left(x_{2}-x_{1}\right)\frac{1+3x_{3}}{2}. \end{array}$$

The proof the proposition follows from the above equality.

**Theorem 9 .**

Let *V*_1/2_ : *S* ^2^ → *S* ^2^ be the evolution operator given by (22). The following statements hold true.

- *ϕ*(*x*) = |*x*_1_ − *x*_2_||*x*_1_ − *x*_3_||*x*_2_ − *x*_3_| is a Lyapunov function.
- Every trajectory converges to the fixed point *C* = (1/3, 1/3, 1/3).

**Proof**

(i) Let *V*_1/2_ be an operator given by (22). It follows from (24) that

(25) $$\begin{array}{c} \phi \left(V_{1/2}\left(x\right)\right)=\phi \left(x\right)\frac{1+3x_{1}}{2}\frac{1+3x_{2}}{2}\frac{1+3x_{3}}{2}. \end{array}$$

On the other hand, we have that

(26) 1+3x121+3x221+3x32 ≤((1+3x1)/2+(1+3x2)/2+(1+3x3)/23)3=1.

Therefore, one has that *ϕ*(*V*_1/2_(*x*)) ≤ *ϕ*(*x*), for any *x* ∈ *S* ^2^. This means that *ϕ* is decreasing a long the trajectory of *V*_1/2_. Consequently, *ϕ* is a Lyapunov function.

(ii) We know that {*ϕ*(*V*_1/2_^(*n*)^(*x*))}_*n*=1_^*∞*^ is a decreasing bounded sequence. Therefore, the limit lim⁡_*n*→*∞*_⁡*ϕ*(*V*_1/2_^(*n*)^(*x*)) = *λ* exists. We want to show that *λ* = 0. Suppose that *λ* ≠ 0. It means that $\overset{-}{{\left\{V_{1/2}^{\left(n\right)}(x)\right\}}_{n=1}^{\infty }}\subset S^{2}\setminus \{l_{1}\;\cup \;l_{2}\;\cup \;l_{3}\}$. Since *λ* ≠ 0, we get that

(27) 1lim⁡n→∞ϕ(V1/2(n+1)(x))ϕ(V1/2(n)(x))lim⁡n→∞(1+3x1(n)21+3x2(n)21+3x3(n)2).

On the other hand, since $\bar{{\left\{V_{1/2}^{\left(n\right)}(x)\right\}}_{n=1}^{\infty }}\subset S^{2}\setminus \{l_{1}\cup l_{2}\cup l_{3}\}$, there exists *ɛ*_0_ such that for any *n* one has that

(28) $$\begin{array}{c} \frac{1+3x_{1}^{\left(n\right)}}{2}\frac{1+3x_{2}^{\left(n\right)}}{2}\frac{1+3x_{3}^{\left(n\right)}}{2}<1-{ɛ}_{0}. \end{array}$$

This is a contradiction. It shows that *λ* = 0.

Therefore, *ω*(*x* ^0^) ⊂ *l*_1_ ∪ *l*_2_ ∪ *l*_3_. We want to show that *ω*(*x* ^0^) = *l*_1_∩*l*_2_∩*l*_3_.

We know that $\left|x_{1}^{\left(n\right)}-x_{2}^{\left(n\right)}\right|\left|x_{1}^{\left(n\right)}-x_{3}^{\left(n\right)}\right|\left|x_{2}^{\left(n\right)}-x_{3}^{\left(n\right)}\right|\overset{n\to \infty }{\to }0$. It follows from (24) that

(29) $$\begin{array}{c} \max\left\{\left|x_{1}^{\left(n\right)}-x_{2}^{\left(n\right)}\right|,\left|x_{1}^{\left(n\right)}-x_{3}^{\left(n\right)}\right|,\left|x_{2}^{\left(n\right)}-x_{3}^{\left(n\right)}\right|\right\}\overset{n\to \infty }{\to }0. \end{array}$$

This means that $\left(x_{1}^{\left(n\right)},x_{2}^{\left(n\right)},x_{3}^{\left(n\right)}\right)\overset{n\to \infty }{\to }\left(1/3,1/3,1/3\right)$. This completes the proof.

### 3.2. Numerical Results on Dynamics of *V*_*α*_

We are going to present some pictures of attractors (an omega limiting set) of the operator *V*_*α*_ : *S* ^2^ → *S* ^2^ given by (15).

In the cases *α* = 0 and *α* = 1, the corresponding operators *V*_0_, *V*_1_ have similar spiral behaviors which reel along the boundary of the simplex [16, 20]. However, one of them moves clockwise and another one moves anticlockwise. In these cases, we have that *ω*_*V*_0__(*x* ^0^)⊂∂*S* ^2^ and *ω*_*V*_1__(*x* ^0^)⊂∂*S* ^2^.

We are interested in the dynamics of the evolution operator *V*_*α*_ while *α* approaches to 1/2 from both left and right sides. In order to see some antisymmetry, we shall provide attractors of *V*_*α*_ and *V*_1−*α*_ at the same time.

**Figure 1:**
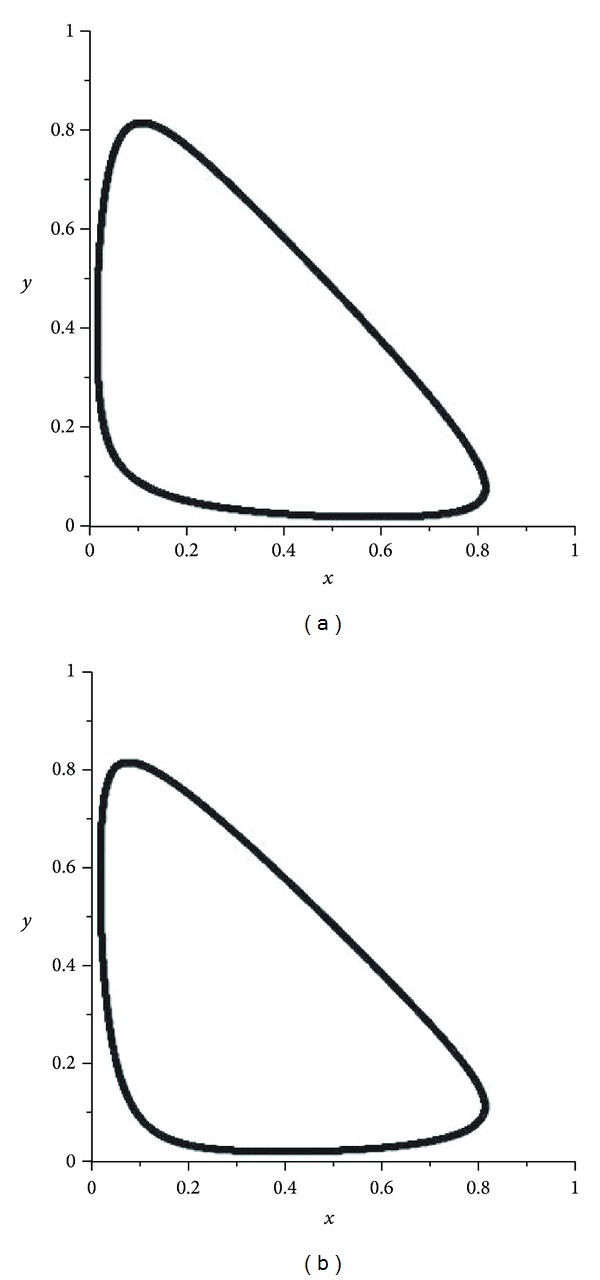
Attractors of *V*_*α*_: *α* = 0.1 and *α* = 0.9.

If *α* is an enough small number, then we can see that the omega limiting sets of operators *V*_*α*_ and *V*_1−*α*_ are separated from the boundary ∂*S* ^2^ (see Figure 1).

**Figure 2:**
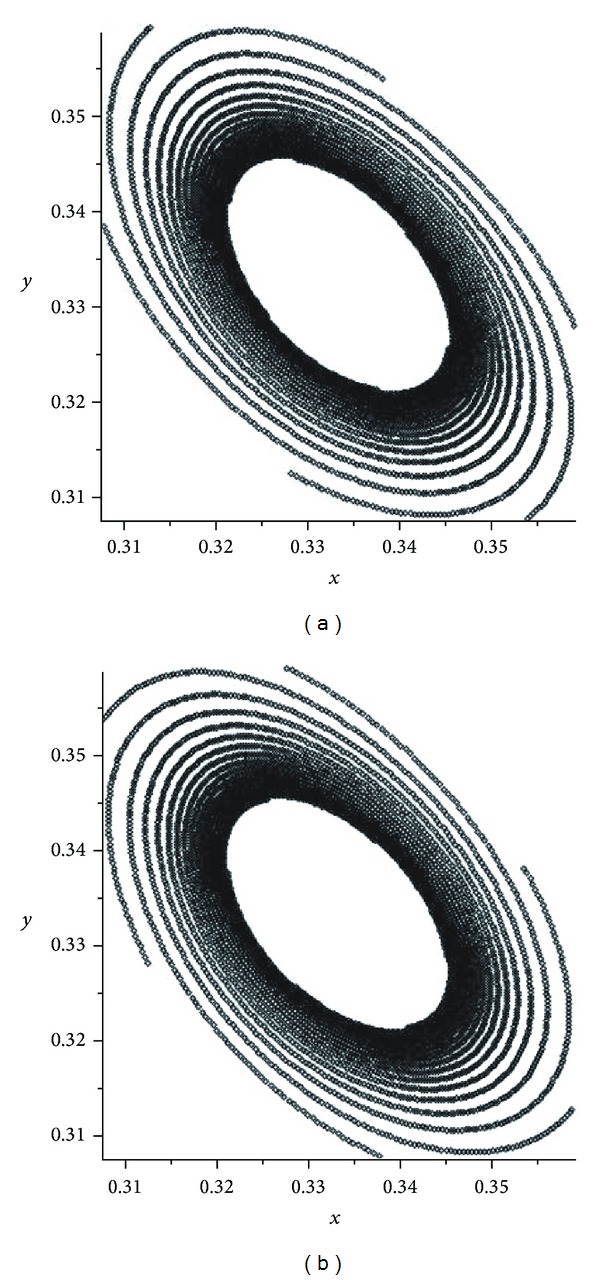
Attractors of *V*_*α*_: *α* = 0.497 and *α* = 0.503.

**Figure 3:**
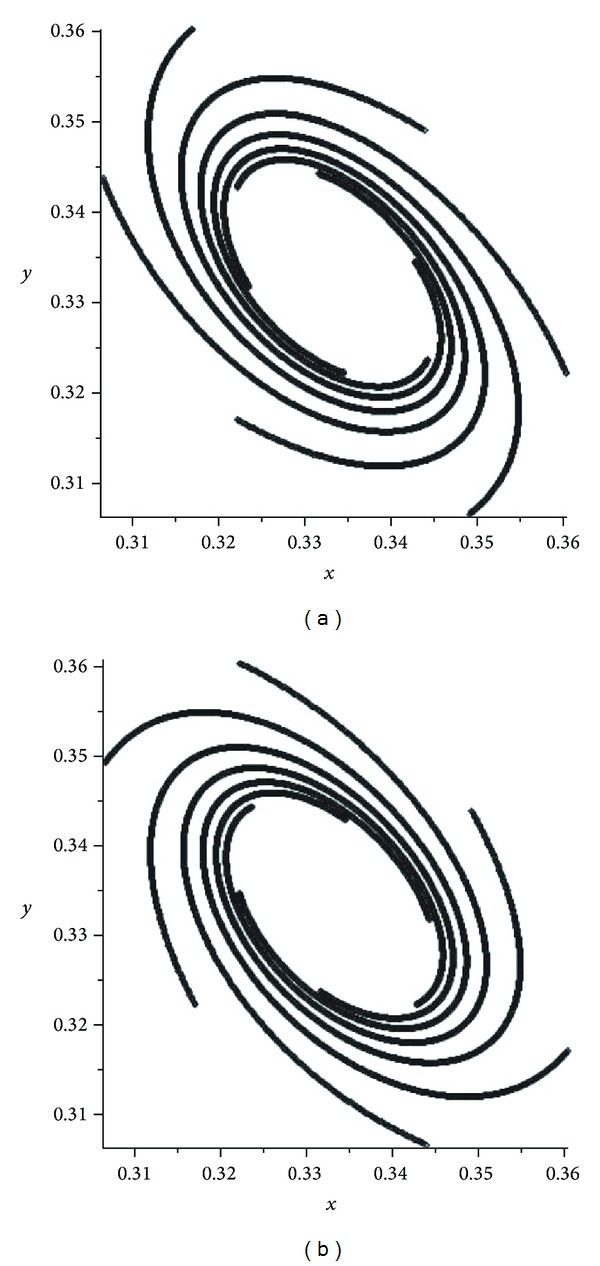
Attractors of *V*_*α*_: *α* = 0.499 and *α* = 0.501.

**Figure 4:**
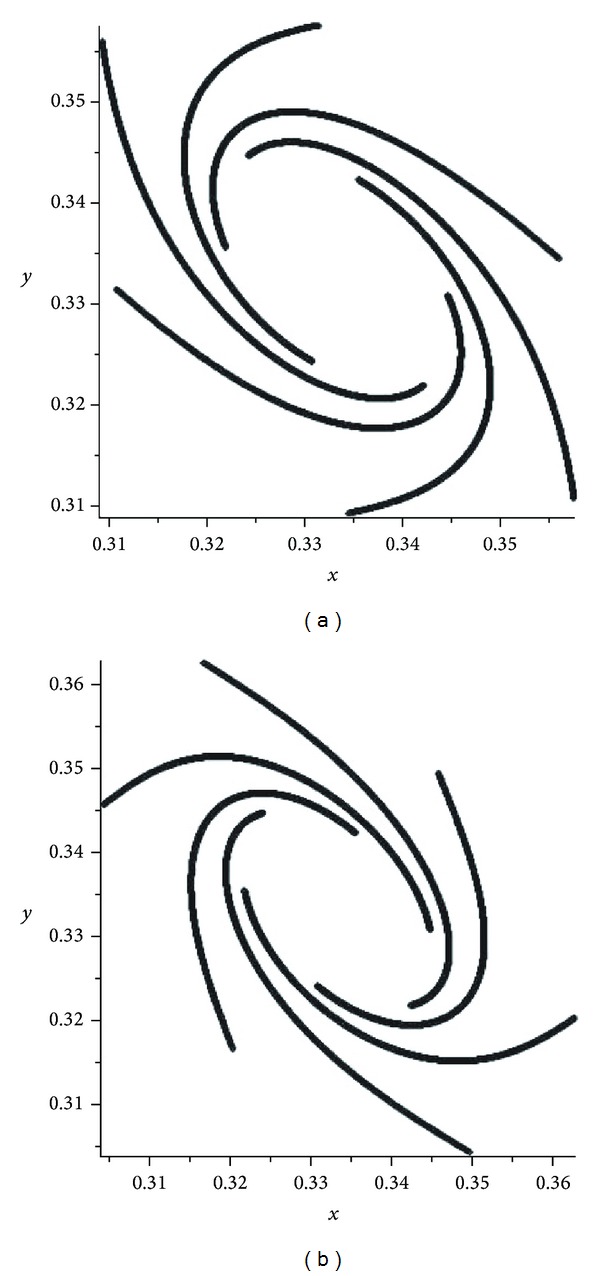
Attractors of *V*_*α*_: *α* = 0.4995 and *α* = 0.5005.

**Figure 5:**
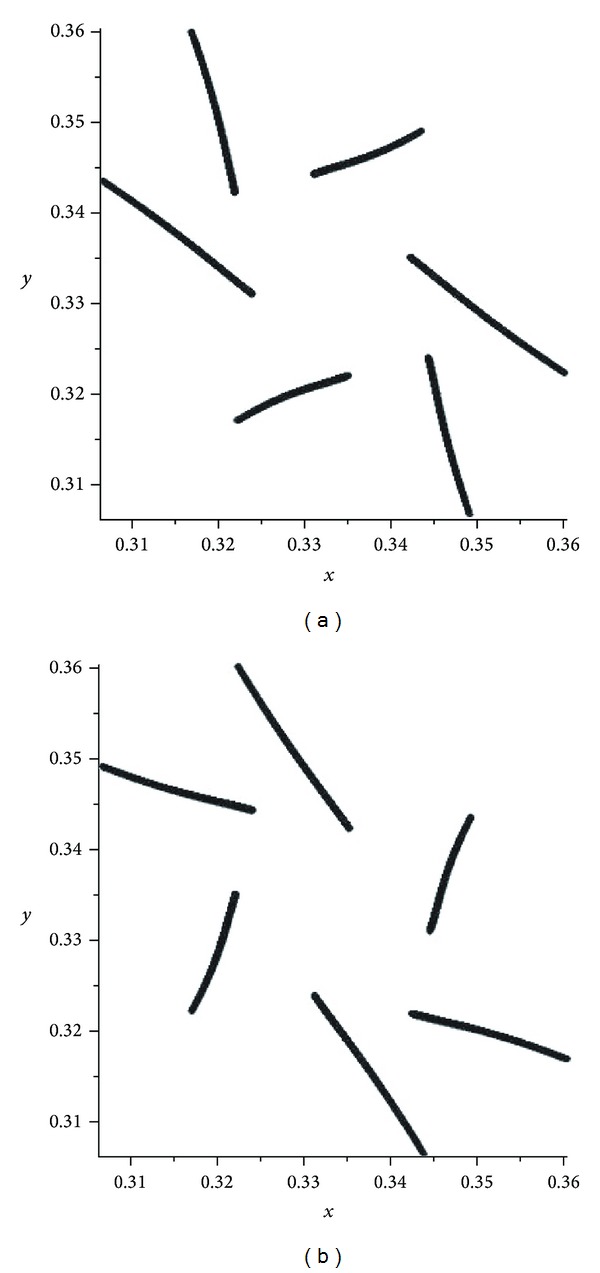
Attractors of *V*_*α*_: *α* = 0.4999 and *α* = 0.5001.

If *α* becomes close to 1/2, then we can see some chaotic pictures. We observe from the pictures (see Figures 2 and 3) that, in the cases *α* and 1 − *α*, the attractors are the same but different from each other by orientations. There are some pictures for the values of *α* = 0.4995,0.4999,0.5005,0.5001 (see Figures 2, 3, 4, and 5). For the evolution operator *V*_*α*_,* the bifurcation point* is *α*_0_ = 1/2 and the influence of the chaotic operators *V*_0_, *V*_1_ would be dismissed. Therefore, the operator *V*_1/2_ becomes regular.

### 3.3. Analytic Results on Dynamics of *W*_*α*_

We are aiming to present some analytic results on dynamics of *W*_*α*_ : *S* ^2^ → *S* ^2^:

(30) $$\begin{array}{c} W_{\alpha }:\{\begin{array}{cc} x_{1}^{\prime }=\left(1-\alpha \right)x_{1}^{2}+2x_{1}x_{2}+\alpha x_{3}^{2}, & \\ x_{2}^{\prime }=\left(1-\alpha \right)x_{2}^{2}+2x_{2}x_{3}+\alpha x_{1}^{2}, & \\ x_{3}^{\prime }=\left(1-\alpha \right)x_{3}^{2}+2x_{3}x_{1}+\alpha x_{2}^{2}. & \end{array} \end{array}$$

As we already mentioned, this operator can be written in the following form: *W*_*α*_ = (1 − *α*)*W*_0_ + *αW*_1_, for any 0 < *α* < 1, where

(31) $$\begin{array}{c} W_{0}:\{\begin{array}{cc} x_{1}^{\prime }=x_{1}^{2}+2x_{1}x_{2}, & \\ x_{2}^{\prime }=x_{2}^{2}+2x_{2}x_{3}, & \\ x_{3}^{\prime }=x_{3}^{2}+2x_{3}x_{1}, & \end{array}\\ W_{1}:\{\begin{array}{cc} x_{1}^{\prime }=x_{2}^{2}+2x_{1}x_{2}, & \\ x_{2}^{\prime }=x_{3}^{2}+2x_{2}x_{3}, & \\ x_{3}^{\prime }=x_{1}^{2}+2x_{3}x_{1}. & \end{array} \end{array}$$

It is clear that *W*_0_ = *V*_0_ is Zakharevich's operator (9) and the operator *W*_1_ is a permutation of the operator which was studied in [11]. By means of methods which were used in [11], we can easily prove the following result.

**Proposition 10 .**

Let *W*_1_ : *S* ^2^ → *S* ^2^ be the evolution operator given as above. Then the following statements hold true.

- The operator *W*_1_ has a unique fixed point *C* = (1/3, 1/3, 1/3) which is attracting.
- The vertexes of the simplex *e*_1_, *e*_2_, *e*_3_ are 3-periodic points.
- *ϕ*(*x*) = *x*_1_^2^ + *x*_2_^2^ + *x*_3_^2^ − 1/3 is a Lyupanov function.
- The operator *W*_1_ is regular in the set int*S* ^2^.

By means of the same methods and techniques which are used for the operator *V*_*α*_, we can prove the following results.

**Proposition 11 .**

Let *W*_*α*_ : *S* ^2^ → *S* ^2^ be the evolution operator given by (30). Then it has a unique fixed point *C* = (1/3, 1/3, 1/3); that is, Fix(*W*_*α*_) = {*C*}. Moreover, one has that

- if $0<\alpha <1-\sqrt{3}/2$, then the fixed point is repelling;
- if $1-\sqrt{3}/2<\alpha <1$, then the fixed point is attracting;
- if $\alpha =1-\sqrt{3}/2$, then the fixed point is non-hyperbolic.

**Theorem 12 .**

Let *W*_*α*_ : *S* ^2^ → *S* ^2^ be the evolution operator given by (30). Then the following statements hold true.

- If $0<\alpha <1-\sqrt{3}/2$, then *ω*(*x* ^0^) ⊂ int*S* ^2^ is an infinite compact set, for any *x* ^0^ ≠ *C*.
- If $1-\sqrt{3}/2\leq \alpha <1$, then *ω*(*x* ^0^) = {*C*}, for any *x* ^0^ ∈ *S* ^2^.

Numerically, we shall see in the next section that the evolution operator *W*_*α*_ : *S* ^2^ → *S* ^2^ given by (30), where $0<\alpha <1-\sqrt{3}/2$, has the following properties.

- The operator *W*_*α*_ is nonergodic.
- The operator *W*_*α*_ exhibits the Li-Yorke chaos.

### 3.4. Numerical Results on Dynamics of *W*_*α*_

We are going to present some pictures of attractors (an omega limiting set) of the operator *W*_*α*_ : *S* ^2^ → *S* ^2^ given by (30).

In the cases *α* = 0 and *α* = 1, the operator *W*_0_ is chaotic and the operator *W*_1_ is regular. Since *W*_*α*_ = (1 − *α*)*W*_0_ + *αW*_1_, the evolution operator *W*_*α*_ gives the transition from the regular behavior to the chaotic behavior. Consequently, we are aiming to find* the bifurcation point* in which* we can see the transition from the regular behavior to the chaotic behavior*.

**Figure 6:**
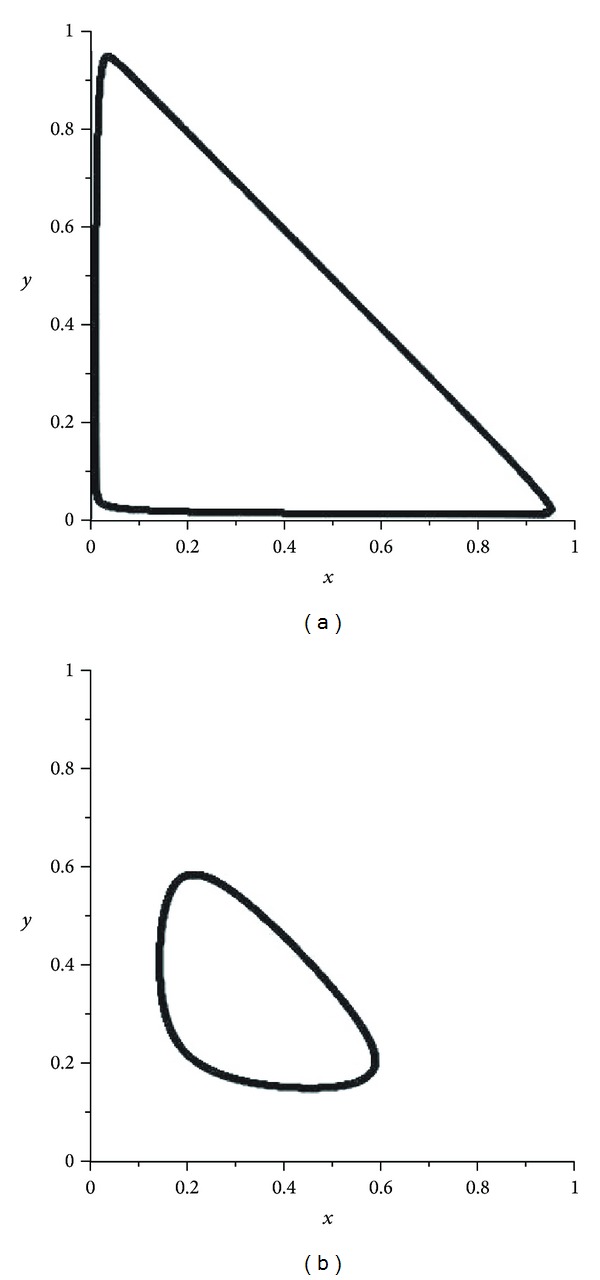
Attractors of *W*_*α*_: *α* = 0.001 and *α* = 0.01.

If *α* is a very small number then attractors of the operator *W*_*α*_ are separated from the boundary of the simplex (see Figure 6). However, the influence of the operator *W*_0_ is still higher and the operator *W*_*α*_ is nonergodic and chaotic.

**Figure 7:**
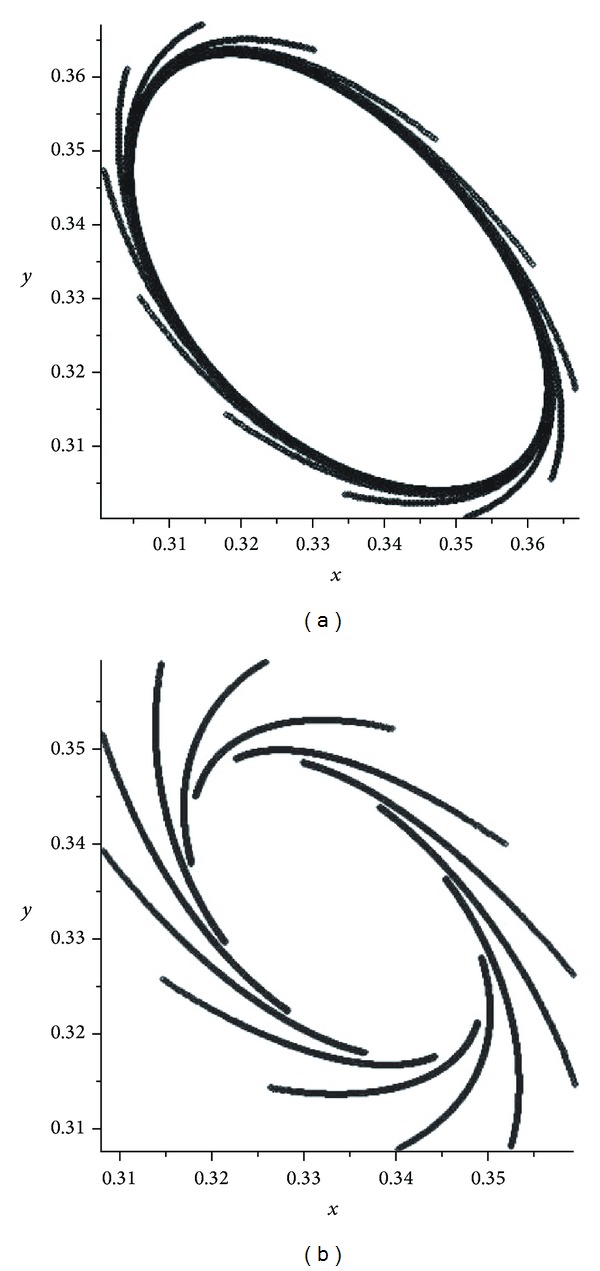
Attractors of *W*_*α*_: *α* = 0.13333 and *α* = 0.1338.

If *α* becomes close to $1-\sqrt{3}/2\;$ (from the left side), then we can see some interesting pictures (see Figure 7). If we continue to increase *α*, then the evolution operator *W*_*α*_ becomes regular (for any $\alpha >1-\sqrt{3}/2$). This means that* the bifurcation point* is $\alpha_{0}=1-\sqrt{3}/2$. Therefore, in order to have a transition from the regular behavior to the chaotic behavior, we need one bifurcation point $\alpha_{0}=1-\sqrt{3}/2$.

## 4. Conclusions

In this paper, we present the mathematical model of the evolution of traits having 3 alleles by mutating the biological environment. We have presented two types of mutations. We have shown that a mutation (a mixing) in the system can be considered as a transition between two different types of systems having Mendelian inheritances. Namely, the first mutation presents the transition between two chaotic biological systems; meanwhile the second mutation presents the transition between regular and chaotic systems.

In the first mutation, we have presented some pictures of attractors of the operator *V*_*α*_ : *S* ^2^ → *S* ^2^ given by (15). In the cases *α* = 0 and *α* = 1, the corresponding operators *V*_0_, *V*_1_ have similar spiral behaviors which reel along the boundary of the simplex. However, one of them moves clockwise and another one moves anticlockwise. In these cases, we had that *ω*_*V*_0__(*x* ^0^)⊂∂*S* ^2^ and *ω*_*V*_1__(*x* ^0^)⊂∂*S* ^2^. If *α* is an enough small number then we observed that the omega limiting sets of operators *V*_*α*_ and *V*_1−*α*_ are separated from the boundary ∂*S* ^2^ (see Figure 1). If *α* becomes close to 1/2 then we had some chaotic pictures. We observed from the pictures (see Figures 2 and 3) that, in the cases *α* and 1 − *α*, the attractors are the same but different from each other by orientations. There are some pictures for the values of *α* = 0.4995,0.4999,0.5005,0.5001 (see Figures 2–5). For the evolution operator *V*_*α*_, the bifurcation point is *α*_0_ = 1/2 and the influence of the chaotic operators *V*_0_, *V*_1_ would be dismissed. Therefore, the operator *V*_1/2_ becomes regular. This means that during the transition between two (in some sense, opposite each other) chaotic systems, at some point of the time, the system should become stable.

**Figure 8:**
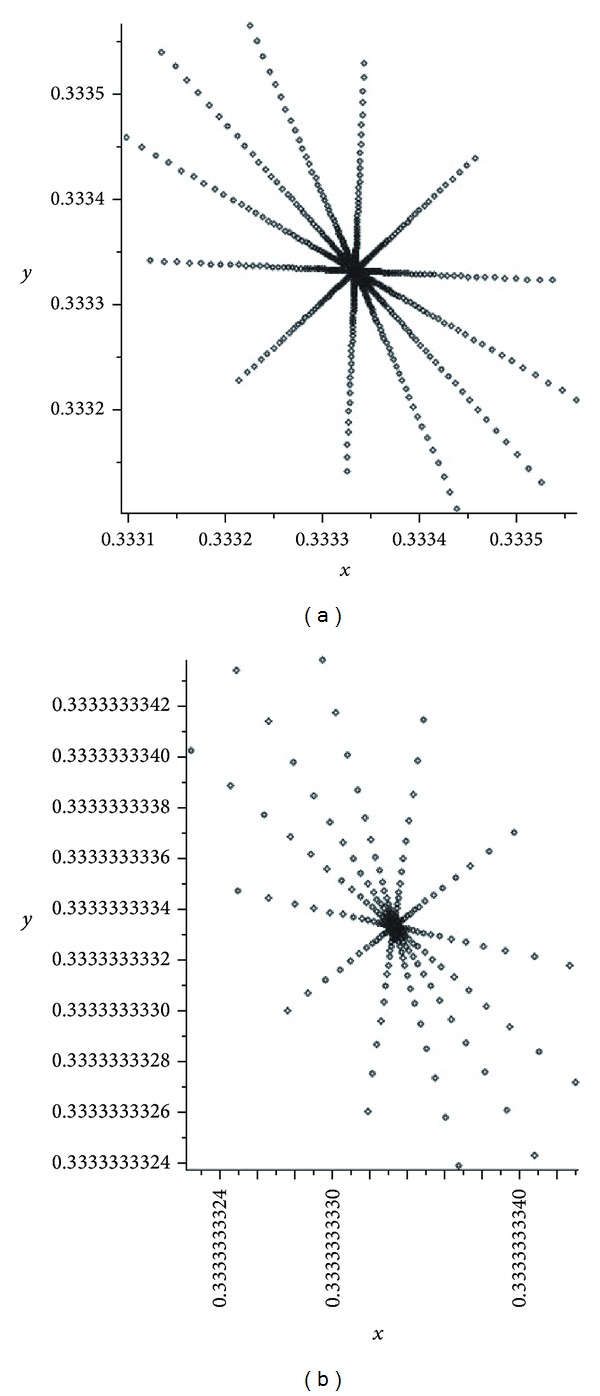
Attractors of *W*_*α*_: *α* = 0.139 and *α* = 0.150.

In the second mutation, we have presented some pictures of attractors of the operator *W*_*α*_ : *S* ^2^ → *S* ^2^ given by (30). In the cases *α* = 0 and *α* = 1, *W*_0_ is chaotic and *W*_1_ is regular. The evolution operator *W*_*α*_ gives the transition from the regular behavior to the chaotic behavior. If *α* is a very small number then attractors of the operator *W*_*α*_ are separated from the boundary of the simplex (see Figure 6). However, the influence of the operator *W*_0_ is still higher and the operator *W*_*α*_ is nonergodic and chaotic. If *α* becomes close to $1-\sqrt{3}/2$ (from the left side), then we can see some interesting pictures (see Figures 7 and 8). If we continue to increase *α*, then the evolution operator *W*_*α*_ becomes regular (for any $\alpha >1-\sqrt{3}/2$). This means that the bifurcation point is $\alpha_{0}=1-\sqrt{3}/2$. Therefore, in order to have a transition from the regular behavior to the chaotic behavior, we need one bifurcation point $\alpha_{0}=1-\sqrt{3}/2$. Since the operator *W*_*α*_ is the convex combination of chaotic (nonergodic) and regular transformations, it is natural to expect the bifurcation scenarios in this evolution. Namely, in order to have a transition from regular to chaotic behavior we have to cross from the bifurcation point. Numerical result *α*⋍0.13397 also confirms the theoretical result about the exact value of bifurcation point. However, the biological plausiblity of this value is unknown for the authors.

In this paper, we have considered two types of mutations of three alleles which occurred with the same probability. It is natural to consider mutations with different probabilities among alleles. In this case, it is expected to have more complicated dynamics in the biological system. The future research is to study the dynamics of the mutated biological system having a single gene with a finite number of alleles.
